# ARPES investigation of the electronic structure and its evolution in magnetic topological insulator MnBi_2+2*n*_Te_4+3*n*_ family

**DOI:** 10.1093/nsr/nwad313

**Published:** 2024-01-08

**Authors:** Runzhe Xu, Lixuan Xu, Zhongkai Liu, Lexian Yang, Yulin Chen

**Affiliations:** State Key Laboratory of Low Dimensional Quantum Physics, Department of Physics, Tsinghua University, Beijing 100084, China; State Key Laboratory of Low Dimensional Quantum Physics, Department of Physics, Tsinghua University, Beijing 100084, China; School of Physical Science and Technology, ShanghaiTech University and CAS-Shanghai Science Research Center, Shanghai 201210, China; School of Physical Science and Technology, ShanghaiTech University and CAS-Shanghai Science Research Center, Shanghai 201210, China; ShanghaiTech Laboratory for Topological Physics, Shanghai 200031, China; State Key Laboratory of Low Dimensional Quantum Physics, Department of Physics, Tsinghua University, Beijing 100084, China; Frontier Science Center for Quantum Information, Beijing 100084, China; Collaborative Innovation Center of Quantum Matter, Beijing 100871, China; School of Physical Science and Technology, ShanghaiTech University and CAS-Shanghai Science Research Center, Shanghai 201210, China; ShanghaiTech Laboratory for Topological Physics, Shanghai 200031, China; Department of Physics, Clarendon Laboratory, University of Oxford, Parks Road, Oxford OX1 3PU, UK

**Keywords:** magnetic topological insulator, MnBi_2_Te_4_, electronic structure, angle-resolved photoemission spectroscopy

## Abstract

In the past 5 years, there has been significant research interest in the intrinsic magnetic topological insulator family compounds MnBi_2+2_*_n_*Te_4+3_*_n_* (where *n* = 0, 1, 2 …). In particular, exfoliated thin films of MnBi_2_Te_4_ have led to numerous experimental breakthroughs, such as the quantum anomalous Hall effect, axion insulator phase and high-Chern number quantum Hall effect without Landau levels. However, despite extensive efforts, the energy gap of the topological surface states due to exchange magnetic coupling, which is a key feature of the characteristic band structure of the system, remains experimentally elusive. The electronic structure measured by using angle-resolved photoemission (ARPES) shows significant deviation from *ab initio* prediction and scanning tunneling spectroscopy measurements, making it challenging to understand the transport results based on the electronic structure. This paper reviews the measurements of the band structure of MnBi_2+2_*_n_*Te_4+3_*_n_* magnetic topological insulators using ARPES, focusing on the evolution of their electronic structures with temperature, surface and bulk doping and film thickness. The aim of the review is to construct a unified picture of the electronic structure of MnBi_2+2_*_n_*Te_4+3_*_n_* compounds and explore possible control of their topological properties.

## Teaser

This article systematically reviews the electronic structures of intrinsic magnetic topological insulators MnBi2+2nTe4+3n (n = 0, 1, 2…), focusing on the evolution of their band structures with temperature, surface/bulk doping, and film thickness.

## INTRODUCTION

In the past two decades, great advances in the field of topological quantum materials have been witnessed [1–7]. Topological quantum phases can be categorized by global topological invariants (such as the Chern number or Thouless–Kohmoto–Nightingale–den Nijs (TKNN) number [8]) rather than the symmetry breaking proposed by Landau. The quantum Hall (QH) trio, including the quantum Hall effect [9], the quantum spin Hall (QSH) effect [10] and the quantum anomalous Hall (QAH) effect [11], are the most representative topological quantum phases in which the quantized conductance that has resulted from the boundary states can be precisely described by using the corresponding topological invariants.

**Figure 1.**
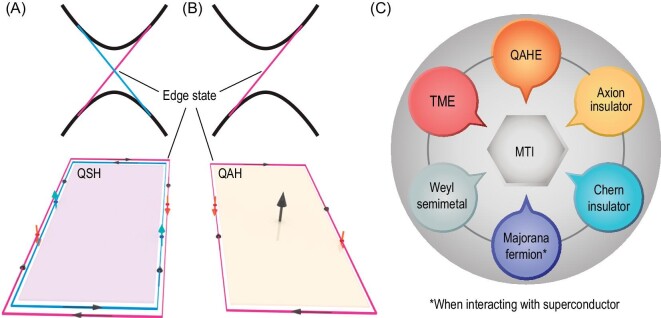
Introduction to the magnetic topological insulator (MTI). (A) and (B) Schematic comparison between 2D topological insulator [quantum spin Hall (QSH) state] and quantum anomalous Hall (QAH) state. (C) Future perspective of MTI as possible parent phase for other quantum states/effects.

The QAH effect shows the quantization (in units of *e*^2^/*h*) of the anomalous Hall conductance under zero magnetic field (sometimes the corresponding insulating state is also referred to as a zero-field Chern insulator) due to the dissipationless chiral edge modes. Although the QAH effect was predicted in the 1980s [11] and the TKNN formula suggests that the Hall conductance in 2D magnetic insulators and semiconductors is always quantized, the experimental realization was absent until 2013 [12] based on the topological insulators (TIs) [13–18]. Due to the band inversion caused by strong spin-orbit couping (SOC), the non-trivial topology of the band structure could be realized in a 2D TI or QSH state (Fig. 1A). After the achievement of the QSH effect, it was proposed that the QAH effect could be realized by introducing magnetism into 2D TIs and establishing the magnetic topological insulator (MTI) phase [19]. In the 2D MTI phase, time-reversal symmetry is broken and the topological surface states (TSSs) are gapped, leading to the QAH phase and the emergence of chiral edge modes (Fig. 1B). MTIs also provide a materials platform to achieve intriguing physical properties or quantum phases such as the topological magnetoelectric effect, axion insulator, Chern insulator state and Weyl semimetal state, and the realization of Majorana fermions (Fig. 1C).

The first attempt to achieve a 2D MTI was made by doping Mn ions into HgTe/CdTe quantum wells [20] but only resulted in quantized Hall conductance under a small magnetic field. Later, based on a similar proposal of introducing magnetic dopants (such as Cr or V) into the 2D TI films (Bi/Sb)_2_(Se/Te)_3_ [21,22], the QAH effect was successfully achieved in Cr-doped (Bi, Sb)_2_Te_3_ thin films in 2013 [12]. Since then, Cr- and V-doped (Bi, Sb)_2_Te_3_ films have been widely studied to investigate the QAH effect [23].

**Figure 2.**
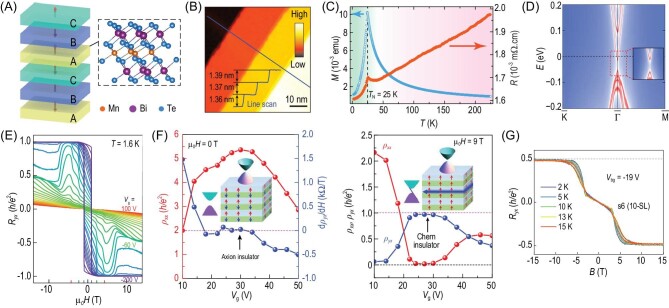
Experimental and theoretical breakthroughs in MnBi_2_Te_4_. (A) Schematic illustration of the crystal and magnetic structures of MnBi_2_Te_4_. (B) Scanning tunneling microscopy mapping of surface topography obtained with *I* = 250 mA and *V* = 1 V (adapted from [40]). (C) Magnetization (with magnetic field applied along the *c*-axis) and resistance as functions of temperature (adapted from [40]). (D) Surface states of the semi-infinite (001) surface showing the gapped TSSs (adapted from [24]). (E) Observation of the QAH effect in a five-septuple layer (SL) exfoliated film (adapted from [29]). (F) Axion insulator and Chern insulator phases in a six-SL exfoliated film [33]. (G) High-Chern-number QH effect without Landau levels in a 10-SL exfoliated film (adapted from [34]).

Despite recent experimental breakthroughs in achieving the QAH phase through magnetic doping, the sample quality is affected by the disorders introduced by dopants, which limits the temperature to establish the QAH effect [50 mK for Cr- and 25 mK for V-doped (Bi, Sb)_2_Te_3_] [12]. Recently, a new class of materials known as intrinsic MTIs has been predicted and realized in MnBi_2+2_*_n_*Te_4+3_*_n_* (where *n* = 0, 1, 2 …) family compounds (Figs 2A and 4A) [24,25–28]. The high magnetic critical temperature (∼25 K) and topological non-triviality of MnBi_2_Te_4_ make it an ideal candidate to achieve the QAH phase. Indeed, the QAH effect was observed in exfoliated five-septuple-layer (SL) MnBi_2_Te_4_ flakes with a relatively high QAH temperature of 1.4 K (Fig. 2E) [29]. Among different experimental platforms to achieve the QAH phase, such as magnetically doped TIs and moiré superlattice systems [30–32], the MnBi_2_Te_4_ system still possesses the highest QAH temperature. Additionally, other remarkable phenomena, such as axion insulator states [33] (Fig. 2F) and high-Chern-number QH states without Landau levels [34] (Fig. 2G), have been observed in antiferromagnetic (AFM) six-SL MnBi_2_Te_4_ flakes and ferromagnetic (FM) 10-SL MnBi_2_Te_4_ flakes, respectively. These experimental breakthroughs make MnBi_2_Te_4_ an ideal platform on which to explore the interplay between magnetism and topology [35].

In the investigation of the 3D MTI and 2D QAH phases, the electronic structure plays a crucial role. The unique topological band structure, such as the inverted bulk bands, TSSs and chiral edge modes, are key indicators of the non-trivial topological nature of MTIs; the size of the inverted bulk gap, the gap size of the TSSs created by exchange interaction and the doping level are important parameters that determine the temperature robustness and transport properties of the QAH phase. Understanding these electronic structure characteristics and key parameters is crucial for potential applications of MTI materials in electronic and spintronic devices.

Angle-resolved photoemission spectroscopy (ARPES) has served as a powerful tool for directly visualizing the electronic structure of topological quantum materials in the momentum space [1,2,4,36,37]. The technique is based on the photoelectric effect [38] and the energy/momentum of the electrons in the samples could be deduced by analysing the energy/momentum of the photoelectrons using conservation laws. The principle of the ARPES instrumentation can be found in Refs [5,39]. For modern ARPES, different photon sources are used, including the vacuum ultraviolet (VUV) laser (*hν* ∼6 to 7 eV) that can provide details of band structure with improved energy and momentum resolutions; and the synchrotron radiation light source (*hν* ∼10 to 200 eV) that can provide photons with tunable energies. In the study of MTIs, both light sources are widely used.

This review will discuss the ARPES investigations carried out on the intrinsic MTI MnBi_2_Te_4_ and its family compounds (MnBi_2_Te_4_)(Bi_2_Te_3_)*_n_* (*n* = 1,2,3…). The primary focus will be on the ARPES evidence that demonstrates the characteristic topological electronic structures of these materials. In particular, the effect of magnetic ordering, the 3D-to-2D crossover in the thin-film limit, as well as the surface/bulk doping on their electronic structures will be discussed in detail, which differentiates the manuscript from previous reviews [44–46].

## ARPES STUDIES OF M${\bf n}$B${\bf i}$_2_T${\bf e}$_4_

**Figure 3.**
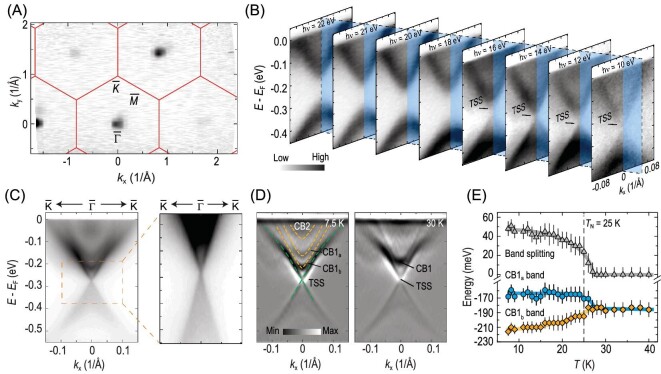
Temperature evolution of the electronic strucure of MnBi_2_Te_4_. (A) Constant-energy contour near the surface Dirac point collected using 100-eV photons at 18 K. (B) ARPES-measured band structure using different photon energies. (C) Band structure measured using laser-ARPES. On the right is the zoom-in plot of the spectra in the dashed rectangle. (D) Comparison between ARPES spectra measured at 7.5 and 30 K. (E) Temperature evolution of the bulk conduction band splitting. Figures are adapted from [40].

Shortly after the theoretical prediction, many synchrotron-based ARPES results were presented to show the basic electronic structure of MnBi_2_Te_4_ [47–51]. Nevertheless, there were controversial and even conflicting results regarding the identification of the TSSs and the magnitude of the surface exchange gap. Figure 3A presents the constant-energy contour of MnBi_2_Te_4_ near 270 meV below *E*_F_ measured using 100-eV photons, which shows point-like features with a periodicity that is consistent with the Brillouin zone. The band dispersions measured at relatively low photon energies are shown in Fig. 3B. The spectra mainly consist of a valence band and a conduction band with a band gap of ∼200 meV, and show a periodic variation with photon energy, suggesting the bulk origin of the band structure measured using relatively high-energy photons [40]. Interestingly, at photon energies of <16 eV, a weak but resolvable feature appears in the band gap, forming a Dirac-like dispersion and showing weak dependence on the photon energy, suggesting its surface origin.

The fine-band dispersion of TSSs can be better visualized using laser-ARPES with improved energy and momentum resolutions. As shown in Fig. 3C, the laser-ARPES spectra are more complex than those collected with synchrotron-based ARPES. Remarkably, there exists a Dirac-like dispersion with a diminishing gap, which can be clearly seen in the zoom-in plot and second derivative of the spectrum (Fig. 3D, left) [40,52,53]. By comparing with the *ab initio* calculations, the Dirac-like band and the parabolic bands were assigned to the TSSs (green dashed curves) and the bulk conduction bands (orange dashed curves, named CB2, CB1_a_ and CB1_b_), respectively.

The observation of the TSSs with a diminishing exchange gap is beyond expectation and remains a controversy in understanding the electronic structure of MnBi_2_Te_4_. There are many scenarios to explain this observation. Here are some examples. (i) Considering the small magnetic anisotropic energy, the interlayer antiferromagnetic interactions may be weaker on the surface than in the bulk, inducing a fluctuating or disordered orientation of surface magnetization, which may recover the time-reversal symmetry and induce a diminishing surface gap [40,52]. (ii) There exist multiple magnetic domains of different magnetization orientations on the sample surface, as confirmed by using magnetic force microscopy [54,55]. These magnetic domains will mediate the surface states together and contribute to the nearly gapless surface state since the in-plane magnetization cannot open the surface exchange gap [56]. (iii) Due to the self-doping effects and/or surface defects, the system prefers a ground state with a nearly gapless surface state [57,58]. Some works also suggest that native point defects play an important role in determining the surface gap [59].

In contrast to the results shown in Fig. 3, many other laser-ARPES experiments also revealed surface exchange gaps of several tens of meV [53,59–61]. The controversial results may be due to the sample variation/inhomogeneity and different data analyses. It is noteworthy that a large exchange coupling between localized spins and the topological bands (100 times larger than the super-exchange interaction) were experimentally revealed recently, which is also controversial to a vanishing exchange coupling on the surface [62]. The rationalization of the contradicting experiments requires further experimental and theoretical investigations.

On the other hand, the temperature evolution of the band structure is crucial for understanding the impact of the antiferromagnetic ordering. As shown in the right of Fig. 3D, the CB1_a_ and CB1_b_ bands merge while the TSSs with diminishing gap remain nearly unchanged above *T*_N_ = 25 K. Figure 3E shows the bottom of the CB1_a_ and CB1_b_ bands as a function of temperature. The band splitting between the CB1_a_ and CB1_b_ bands disappears slightly above *T*_N_, suggesting that the antiferromagnetic ordering indeed influences the electronic structure of the system [40], although the TSSs show negligible temperature dependence.

## MTI (M${\bf n}$B${\bf i}$_2_T${\bf e}$_4_)(B${\bf i}$_2_T${\bf e}$_3_)*_n_* (***n***** = 1,2,3**, …) FAMILY

**Figure 4.**
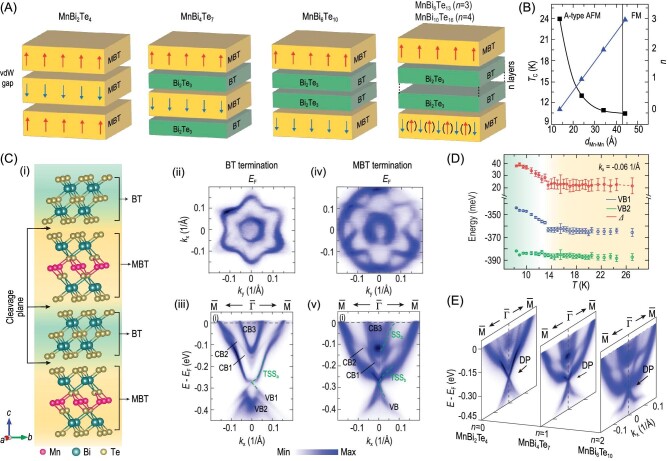
ARPES investigation of (MnBi_2_Te_4_)(Bi_2_Te_3_)*_n_* (*n* = 0, 1, 2) superlattices. (A) Schematic illustration of the crystal and magnetic structures of (MnBi_2_Te_4_)(Bi_2_Te_3_)*_n_* compounds consisting of alternating Bi_2_Te_3_ (BT) and magnetic MnBi_2_Te_4_ (MBT) blocks (adapted from [41]). (B) *T*_c_ versus *d*_Mn__–__Mn_ (the interlayer distance between the adjacent Mn–Mn layers) and *n* versus *d*_Mn__–__Mn_ in (MnBi_2_Te_4_)(Bi_2_Te_3_)*_n_* (*n* = 1, 2, 3 and 4) (adapted from [42]). (C) (i) Schematic illustration of crystal structure (two-unit cells) of MnBi_4_Te_7_; (ii) constant-energy contour and (iii) band dispersions on the BT termination. (iv) and (v) Same as (ii) and (iii), but on the MBT termination (adapted from [43]). (D) Temperature evolution of the electronic structure of BT-terminated MnBi_4_Te_7_. The energy positions of VB1 and VB2 bands together with their energy difference are plotted as functions of temperature (adapted from [43]). (E) Electronic structure on the MBT termination of MnBi_2_Te_4_, MnBi_4_Te_7_ and MnBi_6_Te_10_ (adapted from [40,42,43]).

The van der Waals interaction between MnBi_2_Te_4_ layers allows the insertion of other layers, such as Bi_2_Te_3_ layers, to form a series of superlattices (MnBi_2_Te_4_)(Bi_2_Te_3_)*_n_* (*n* = 0,1,2, …) (Fig. 4A) [41,42,63,64]. By controlling the ratio of the MnBi_2_Te_4_ (MBT) and Bi_2_Te_3_ (BT) layer numbers, the coupling between adjacent MnBi_2_Te_4_ layers could be systematically tuned: the magnetic coupling is weakened with increased distance between MnBi_2_Te_4_ layers, which leads to the reduction in the magnetic ordering temperature (Fig. 4B). With *n* > 3, the ground state of the compound switches from an A-type AFM state to an FM state (Fig. 4A and B). Such flexibility makes this series of compounds an ideal platform for investigating the interplay between magnetism, topology and electronic structure in intrinsic MTIs, as exemplified by MnBi_4_Te_7_ in Fig. 4.

After cleavage, MnBi_4_Te_7_ shows two types of surface terminations: MBT and BT [see Fig. 4C(i)]. Laser-ARPES was used to identify the topological electronic structures including the characteristic TSSs on both terminations. While both terminations show a petal-like hexagonal Fermi surface together with a small electron pocket around the ${\mathrm{\bar{\Gamma }}}$ point, the MBT termination exhibits an additional large circular electron pocket [see Fig. 4C(ii) and (iv)]. The bulk bands [conduction bands (CB1, CB2 and CB3) and valance bands (VB1 and VB2)] along $\bar{M} - {\mathrm{\bar{\Gamma }}} - \bar{M}$ do not change with surface termination, while the surface states (TSS_a_ on BT termination and TSS_b_ and SS_b_ on MBT termination) show distinctive dispersions on the two terminations [Fig. 4C(iii) and (v)]. Similarly to the situation in Bi_2_Te_3_, the bulk CB and VB are contributed by Te-*p_z_* and Bi-*p_z_* orbitals with opposite parities, which confirms the non-trivial topology of MnBi_4_Te_7_. The bulk band gap size is estimated to be ∼100 meV, which is comparable to the bulk band gap in MnBi_2_Te_4_ [40,43].

Remarkably, on both terminations, within the energy resolution of laser-ARPES experiments (2.5 meV), the TSSs in the bulk gap are found to be nearly gapless, which is apparently different from the calculated results that predict TSSs with an energy gap of ∼28 meV. The observation, however, is similar to the situation in the sister compound MnBi_2_Te_4_ with a diminishing surface gap. Furthermore, due to the small energy difference between various magnetic configurations in MnBi_4_Te_7_, many nanosized magnetic domains of different magnetization orientations can coexist on the sample surface, forming a complicated surface magnetic structure other than the theoretically proposed A-type AFM configuration. In this scenario, the TSSs remain gapless in magnetic domains that respect the time-reversal symmetry.

The temperature-dependent measurements of both bulk bands and TSSs show an intriguing difference across the magnetic phase transition: while the bulk states show a clear reduction in band splitting (Fig. 4D), the TSSs remain gapless. This difference is also similar to the results of MnBi_2_Te_4_ and indicates a complex influence of magnetism and interlayer coupling on the topological electronic structure. The results on the *n* = 1 sample are consistent with other ARPES results [65–68].

The ARPES results on *n* = 2 samples are more complicated as three types of terminations could be found. However, the key findings are similar. An unexpected but universal gapless Dirac cone is observed on the MBT surfaces, indicating an altered magnetic structure near the surface (Fig. 3E). On other terminations, the band dispersion of the surface states is dominated by the top surface, remains nearly gapless and is sensitive to different stackings of the underlying MBT and BT layers [68].

For *n* = 3 samples, there has been an ARPES report unveiling a massive Dirac gap (∼28 meV) at the MBT-termination surface that decreases monotonically with increasing temperature and closes right at the Curie temperature, thereby representing the first smoking-gun spectroscopic evidence of a magnetization-induced topological surface gap among all known magnetic topological materials [69]. On other terminations, gapless Dirac cones could be found. The observed difference between the *n* = 3 and *n* = 0, 1 and 2 counterparts can be attributed to the variation in the FM order for *n* = 3 and the A-AFM order for the *n* = 0, 1 and 2 samples. This difference in magnetic order results in a more effective magnetic ordering on the TSSs for *n* = 3 samples, leading to the formation of a gapped Dirac cone.

## BULK AND SURFACE DOPING OF M${\bf n}$B${\bf i}$_2_T${\bf e}$_4_

**Figure 5.**
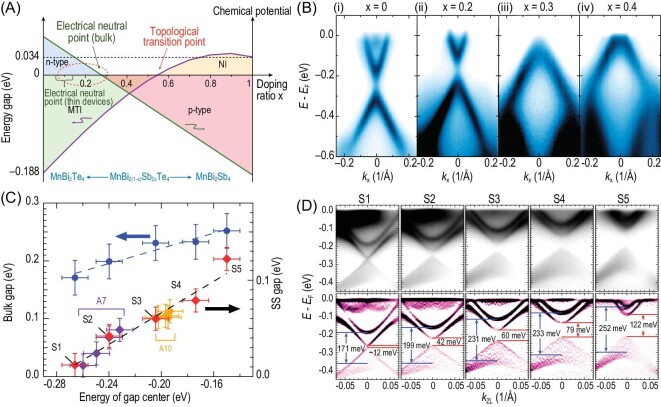
Phase diagram and existence of Dirac gap in Mn(Bi_1__–_*_x_*Sb*_x_*)_2_Te_4_. (A) Phase diagram of Mn(Bi*_x_*Sb_1__–_*_x_*)_2_Te_4_, showing transition of n-type to p-type at *x* = 0.29 and topological phase transition at *x* = 0.55. (B) Photoemission intensity of Mn(Bi_1__–_*_x_*Sb*_x_*)_2_Te_4_ along the high symmetry direction with *x* = 0, 0.2, 0.3 and 0.4, showing transition from n-type to p-type. (C) Energy of gap center as a function of the bulk gap at Z (left) and the surface state gap (right) with *x* between 0 and 0.1. (D) Raw (top) and second derivative (bottom) ARPES spectra for five samples with different carrier concentrations ordered by the energy of the gap center. Red/blue lines: surface state/bulk gap. (A) and (B) are adapted from [49] and (C) and (D) are adapted from [73].

The electronic structure of MnBi_2_Te_4_ could be systematically tuned via bulk and surface doping. The doping to the stoichiometric compound would effectively modify its electronic structure towards the desired one. In the MTI case, the goal would be the QAH phase with low carrier concentrations, topological non-trivial band structure and high critical temperature. In previous research, such a purpose was achieved by isovalent substitution of Sb with Bi in Cr-doped (Bi, Sb)_2_Te_3_, which drives the compound to the charge-neutral point before tuning the fine position of E_F_ with gating [12]. The Sb dopant serves as an effective hole dopant while keeping the high quality of the compound. A similar strategy is also adopted in the intrinsic MTI MnBi_2_Te_4_ in which Sb-substituted MnBi_2_Te_4_ has been systematically investigated (Fig. 5). Similarly to the (Bi, Sb)_2_Te_3_ case, Sb doping effectively tunes the carrier concentration and leads to the transition of n-type to p-type from MnBi_2_Te_4_ to MnSb_2_Te_4_ (Fig. 5A), as evidenced by using both transport and ARPES experiments (Fig. 5B) [49,70]. Topological phase transition is predicted by the first-principle calculations of Mn(Bi_0.7_Sb_0.3_)_2_Te_4_, which suggests that MnSb_2_Te_4_ is a topological trivial insulator [49]. However, there have been reports proposing that MnSb_2_Te_4_ and MnSb_4_Te_7_ are FM TIs [71,72].

An interesting finding is that, with Sb substitution, a Dirac gap opens immediately and the gap size increases monotonically with the Sb-doping level (Fig. 5C and D) [73]. Such an experimental finding is consistent with the observation of the Dirac gap in Cr-doped Bi_2_Se_3_ films with its size tunable by the chemical potential via Mg doping [74] and could be interpreted as the localization effect from the increase in impurities. Such an observation may contribute to the physical interpretation of the TSS gap observed in the ARPES measurement.

On the other hand, the electronic structure could be effectively modified by *in situ* surface potassium (K) adsorption in the ARPES measurements. This method can help identify different components of the TSSs [75–77] and neutralize the undesired trivial surface states, which can realize a minimal MTI in MnBi_2_Te_4_ [78].

**Figure 6.**
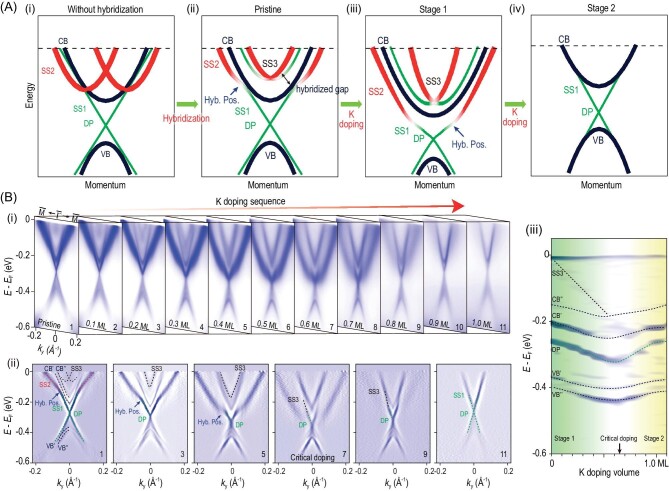
ARPES study of surface K-doped MnBi_2_Te_4_ samples. (A) (i) Schematic illustration of the band structure of MnBi_2_Te_4_; the Rashba-type surface state (SS2) and the TSS (SS1) do not hybridize; (ii) the hybridization between SS1 and SS2 rearranges surface states and generates the Rashba-type SS3; (iii) band structure of MnBi_2_Te_4_ with slight surface K doping; (iv) band structure of MnBi_2_Te_4_ after massive K doping. (B) (i) Evolution of the band structure along${\mathrm{\ \bar{\Gamma }}}-{\mathrm{\bar{M}}}$ with surface K coverage; (ii) representative curvature plots of the spectra in (i); the splitting of conduction (CB′, CB′′) and valence (VB′, VB′′) bands are due to the AFM order; (iii) curvature plot of the image formed by stacking the energy distribution curves (EDCs) at *k*_y_ = 0 from those in (i); energy bands, the two-stage crossover doping process and the critical doping are denoted. Figure adapted from [75].

Figure 6A provides a schematic of the major findings. The TSS in MnBi_2_Te_4_ is heavily hybridized with a Rashba-type surface state (RSS) [see Fig. 6A(i) and (ii)], which could be effectively manipulated through a two-stage crossover process of continuous surface K doping: in Stage 1, the hybridization position shifts down in synchronization with the SS3 [Fig. 6A(iii)] while, in Stage 2, the RSS gradually disappears and the overall band structure approaches a minimal MTI electronic structure with only TSS and inverted bulk bands near *E*_F_ [Fig. 6A(iv)].

Figure 6B shows a systematic evolution of the band dispersions along ${\mathrm{\bar{\Gamma }}}$–${\mathrm{\bar{M}}}$ with sequential K doping. The energy bands first shift towards higher binding energies then return back towards *E*_F_, showing a non-monotonic two-stage doping evolution (upward/downward shift of the Fermi level). Due to their surface nature, SS1, SS2 and SS3 are more sensitive to the surface doping than the bulk bands (CB′/CB″ and VB′/VB″). The two-stage evolution process is separated by a critical K-doping level at which the hybridization between SS1, SS2 and SS3 is not resolvable. Eventually, at a heavy doping level, the topological trivial surface states are eliminated and the TSSs and inverted bulk bands form the minimal MTI band structure.

According to the detailed core level (Te 4d, Bi 5d and the K 3p) analysis, the microscopic mechanism of K doping was interpreted as a two-stage electrochemical reaction process. At Stage 1, a few K atoms are adsorbed on the MnBi_2_Te_4_ surface, which raises the Fermi level due to the charge transfer from K atoms to MnBi_2_Te_4_. At Stage 2, K atoms continue to accumulate, resulting in significant interaction between K^+^, which tends to drive them into segregated K clusters. The enhanced surface roughness therefore suppresses the topologically trivial surface states. The clustering of K atoms also reduces the charge transfer to the MnBi_2_Te_4_ surface. Furthermore, the massive K doping allows K−Te−Bi alloying. The reduced-Bi-containing alloy extracts electrons from the surface of MnBi_2_Te_4_, causing a decrease in free carriers on the surface and a downward shift of the Fermi level.

The K-doping ARPES measurements not only provide a simplified electronic structure and clear band assignments of MnBi_2_Te_4_, but also lead to a ‘passivated’ layer with minimal topological electronic structure. This allows future investigation of the quantum transport behavior and the potential for device application with intrinsic MTIs.

## ELECTRONIC STRUCTURE OF M${\bf n}$B${\bf i}$_2_T${\bf e}$_4_ THIN FILMS

In addition to the diminishing surface gap, there are other peculiarities in the electronic structure of MnBi_2_Te_4_. First, there is a kink-like structure in the dispersion of TSSs. Second, the topological surface band becomes broadened and weakened when approaching the Fermi level. Third and importantly, while the transport breakthroughs were all realized in thin films of several SLs, the measured electronic structure of the thin films was clearly different from that of bulk samples [79]. To understand these strange observations, it is crucial to investigate the electronic structure of MnBi_2_Te_4_ and its evolution using film thickness.

The films of MnBi_2_Te_4_ can be either grown by using a molecular-beam-epitaxy (MBE) system or exfoliated from the bulk sample. However, the size of the exfoliated films is usually <10 microns, which limits ARPES measurements. Alternatively, high-quality large-sized thin films of MnBi_2_Te_4_ can be synthesized using a MBE system. The characterization of the thin films using reflected high-energy electron diffraction and scanning tunneling microscopy confirmed the high quality of the films [80]. Similar to the surface of the bulk sample, there exist considerable defects mainly from Mn atoms occupying the Bi sites (Mn–Bi antisite defects) in the thin films [26]. Prominently, transport measurements in the thin films revealed a quantized anomalous Hall effect (AHE) in the five-SL film [81], consistently with the results in the exfoliated five-SL films [29].

**Figure 7.**
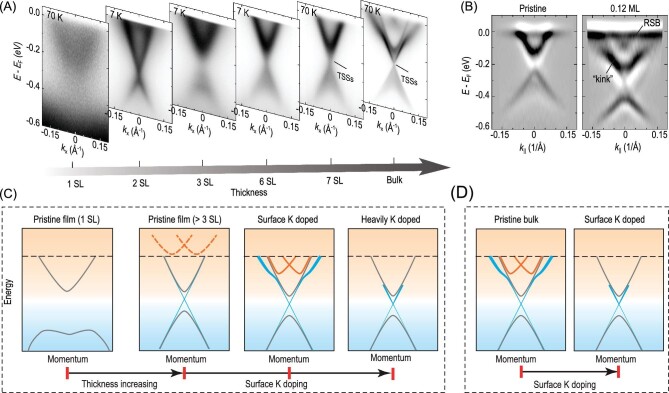
Evolution of the electronic strucure of MnBi_2_Te_4_ films. (A) Thickness-dependent band structure of MnBi_2_Te_4_ films. (B) Comparison between the second derivative of ARPES spectra of pristine and K-doped five-septuple layer (SL) film. (C) Schematic illustration of the band structure of MnBi_2_Te_4_ films and (D) bulk sample with and without surface K doping. Figure adapted from [80].

The electronic structures of MnBi_2_Te_4_ thin films grown by using MBE were systematically investigated by using ARPES [79,80,82–87]. Figure 7 shows the results collected using a 7-eV laser. The measured band structure shows a clear evolution with film thickness. In the one-SL film, the band structure consists of an M-like valence band and a parabolic conduction band with a band gap of ∼300 meV between them. Interestingly, the band gap shrinks in the two-SL film and then reopens in the three-SL film (Fig. 7A). Above three SLs, there are in-gap states that emerge and gradually evolve into the TSSs in the thick films. No clear change in the band structure was observed above four SLs except for the increasing spectral weight of the in-gap states. *Ab initio* calculations suggest different Chern numbers of odd- and even-number SL films: while the Chern number changes from 0 in one-SL film to 1 in three-SL film, the Chern number of all the even-number SL films is 0 [80,88].

The electronic structure of the films is drastically different from that of the bulk sample. Although the topological surface band also shows a diminishing gap, it does not exhibit a kink-like structure. Instead, it merges into the bulk conduction band. Besides, the Fermi level of the films differs from that of the bulk sample. Therefore, surface doping of alkali metals was adopted to raise the Fermi level of the films and modify the surface condition. Through surface K doping, the TSSs gradually separate from the bulk conduction band and a kink-like structure emerges in the dispersion of the TSSs. At the doping level of the 0.12 monolayer of K, the band structure becomes similar to that of the bulk sample, despite the broadened spectra, as shown in Fig. 7B. Simultaneously, an extra band crossing appears near the Fermi level, which shifts synchronously with the kink-like structure.

The synchronized response of the band crossing and the kink-like feature suggest a common origin, as schematically shown in Fig. 7C. There exists an unoccupied Rashba split band (RSB) in the films that are thicker than three SLs. With surface K doping, the RSB shifts towards high-binding energies and hybridizes with the TSSs, inducing the kink-like structure. This scenario is confirmed by using surface-doping-dependent measurement on the bulk sample, which also demonstrates a lock-in evolution of the RSB and the kink-like structure. At heavy doping, both the RSB and the kink disappear, leaving a band structure with only a bulk valence and a conduction band as well as the TSSs connecting them. The similar evolution of the thin films and bulk sample suggests that the drastic difference in the band structure between the pristine film and the bulk sample may stem from the surface condition, which can be effectively tuned by surface K doping.

The RSB observed in both the films and the bulk sample resembles that in the Bi_2_Se_3_, which is related to the quantum well states formed after surface doping. Simulations based on a four-band model of TIs suggest that the RSB originates from the quantum confinement effect, resembling the doping evolution of the band structure of Bi_2_Se_3_ [89,90].

The observation of the RSB and its interaction with the topological surface state is not trivial. First, the RSB contributes significant carriers in the system, which will play a role in the transport properties. Second, it provides a platform for realizing the Rashba ferromagnet considering the surface ferromagnetism of MnBi_2_Te_4_. As proposed by theory, the SOC and ferromagnetism may open a gap at the band crossing point of the RSB [96,97]. Recent ARPES experiments observed the gap opening of the Kramer point of the RSB induced by AFM ordering [61,98], which may be an alternative mechanism for the QAH effect other than the exchange gap at the Dirac point of the TSSs.

## SUMMARY AND PERSPECTIVE

Despite extensive research, the existence and magnitude of the exchange energy gap of the TSSs in MnBi_2_Te_4_ remain controversial. Table 1 summarizes ARPES-measured exchange gaps of the TSSs of (MnBi_2_Te_4_)(Bi_2_Te_3_)*_n_* compounds. It is clear that the conclusion regarding gapped or gapless TSSs strongly relies on the experimental instrument and data analysis. To reach a consensus, samples with higher quality, better energy and momentum resolution in measurement, lower sample temperature, single magnetic domain measurements and direct measurement on exfoliated thin-film samples are required for future ARPES measurements.

**Table 1.** Exchange gap values of MnBi_2+2_*_n_*Te_4+3_*_n_* (where *n* = 0, 1, 2 …) family measured by using ARPES.

Materials	Band gap (meV)	Termination/thickness	Literature	Light source
MnBi_2_Te_4_	Nearly gapless	MBT	Chen *et al.* [40]. Hao *et al.* [52]. Swatek *et al.* [91]	Laser
	60 (10 K)		Estyunin *et al.* [61]	
	14 (8 K) 12 (40 K)		Li *et al.* [53]	
	15–65 (10–16 K)		Shikin *et al.* [60]	
	85 (5–300 K)		Lee *et al.* [50]	He lamp
	150 (15 K)		Yan *et al.* [92]	
	100 (10∼80 K)		Vidal *et al.* [48]	Synchrotron
MnBi_4_Te_7_	Gapless	MBT and BT	Xu *et al.* [43]	Laser
	Gapless	MBT	Hu *et al.* [68]	Synchrotron
	Gapless	MBT	Gordon *et al.* [93]	
	100	BT_1_		
	Gapped	MBT and BT_1_	Vidal *et al.* [67]	
MnBi_6_Te_10_	∼30	MBT	Jo *et al.* [94]	Laser
	Gapless	BT_1_ and BT_2_		
	Gapless	MBT, BT_1_ and BT_2_	Hu *et al.* [68]	
	28	MBT	Tian *et al.* [95]	He lamp
	Gapless	BT_1_ and BT_2_		
	Gapped	MBT, BT_1_ and BT_2_	Vidal *et al.* [67]	Synchrotron
	Gapless	MBT and BT_2_	Gordon *et al.* [93]	
	140	BT_1_		
MnBi_8_Te_13_	Gapless	MBT, BT_2_ and BT_3_	Hu *et al.* [42]	Synchrotron
	105	BT_1_		
Thin-film MnBi_2_Te_4_	140/60	1 SL/2SL	Xu *et al.* [80]	Laser
	Gapless	≥3 SL		
	120	1 SL	Gong *et al.* [79]	He lamp
	Gapless	≥2 SL		
	>800	1 SL	Trang *et al.* [86]	Synchrotron
	300	2 SL		
	70	3 SL		
	70	5 SL		

On the other hand, the manipulation of the electronic structure via bulk and surface doping offers a promising approach to improve the topological properties of (MnBi_2_Te_4_)(Bi_2_Te_3_)*_n_*. This method effectively eliminates the trivial surface states and allows the tuning of system magnetization through the insertion of Bi_2_Te_3_/MnTe layers or bulk substitution of Bi with Sb. Moreover, the strong interaction of TSSs with the RSB provides new insights into understanding the novel topological properties, such as the anomalous Hall effect and QAH effect in MnBi_2_Te_4_. High-resolution ARPES will continue to provide crucial information in the exploration and application of the novel properties of the MnBi_2+2_*_n_*Te_4+3_*_n_* (where *n* = 0, 1, 2 …) family MTIs.
